# Strong interband Faraday rotation in 3D topological insulator Bi_2_Se_3_

**DOI:** 10.1038/srep19087

**Published:** 2016-01-11

**Authors:** L. Ohnoutek, M. Hakl, M. Veis, B. A. Piot, C. Faugeras, G. Martinez, M. V. Yakushev, R. W. Martin, Č. Drašar, A. Materna, G. Strzelecka, A. Hruban, M. Potemski, M. Orlita

**Affiliations:** 1 Institute of Physics, Charles University, Ke Karlovu 5, CZ-121 16 Praha 2, Czech Republic; 2 Laboratoire National des Champs Magnétiques Intenses, CNRS-UJF-UPS-INSA, 25, avenue des Martyrs, 38042 Grenoble, France; 3 Department of Physics, SUPA, Strathclyde University, G4 0NG Glasgow, UK; 4 Ural Federal University and Institute of Solid State Chemistry of RAS, Ekaterinburg, 620002, Russia; 5 Institute of Applied Physics and Mathematics, Faculty of Chemical Technology, University of Pardubice, Studentská 84, 532 10 Pardubice, Czech Republic; 6 Institute of Electronic Materials Technology, ul. Wolczynska 133, PL 01-919 Warsaw, Poland

## Abstract

The Faraday effect is a representative magneto-optical phenomenon, resulting from the transfer of angular momentum between interacting light and matter in which time-reversal symmetry has been broken by an externally applied magnetic field. Here we report on the Faraday rotation induced in the prominent 3D topological insulator Bi_2_Se_3_ due to bulk interband excitations. The origin of this non-resonant effect, extraordinarily strong among other non-magnetic materials, is traced back to the specific Dirac-type Hamiltonian for Bi_2_Se_3_, which implies that electrons and holes in this material closely resemble relativistic particles with a non-zero rest mass.

The recently emerged class of topological insulators (TIs)^1^,^2^,^3^ comprises materials with specific Dirac-type surface states, which continuously connect otherwise well-separated conduction and valence bands. The existence of these intriguing surface states is encoded in the specific bulk electronic band structures, which combines band inversion with time-reversal symmetry. The sensitivity to perturbations breaking the time-reversal symmetry makes TIs, and in particular their surface states, natural targets of Faraday rotation experiments^4^,^5^,^6^,^7^, allowing us, among other things, to trace the crossover from the topological to normal state of matter. Opening the band gap in the TI surface states should be, at subgap photon energies, manifested by a Faraday angle determined only by the fine structure constant *α*^8^,^9^. Other universal Faraday rotation effects have been proposed for Landau-quantized surface states of TIs^10^,^11^. These become analogous to predictions for other quantum Hall systems, including graphene and electron/hole gases in conventional 2D semiconductor heterostructures^12^, which have already been tested in the very first experiments^13^,^14^,^15^.

In this paper, we report on a strong Faraday rotation in the well-known Bi_2_Se_3_ 3D topological insulator. We show that the observed effect appears due to interband excitations in bulk, from the valence to the partially filled conduction band. The strength of the rotation, expressed in terms of the Verdet constant, is found to be extraordinary large for a non-magnetic material. This is related to the specific bulk electronic band structure of Bi_2_Se_3_, which implies that charge carriers closely resemble massive relativistic particles, with the spin-splitting large and equal for electrons and holes.

## Results

**Figure 1:**
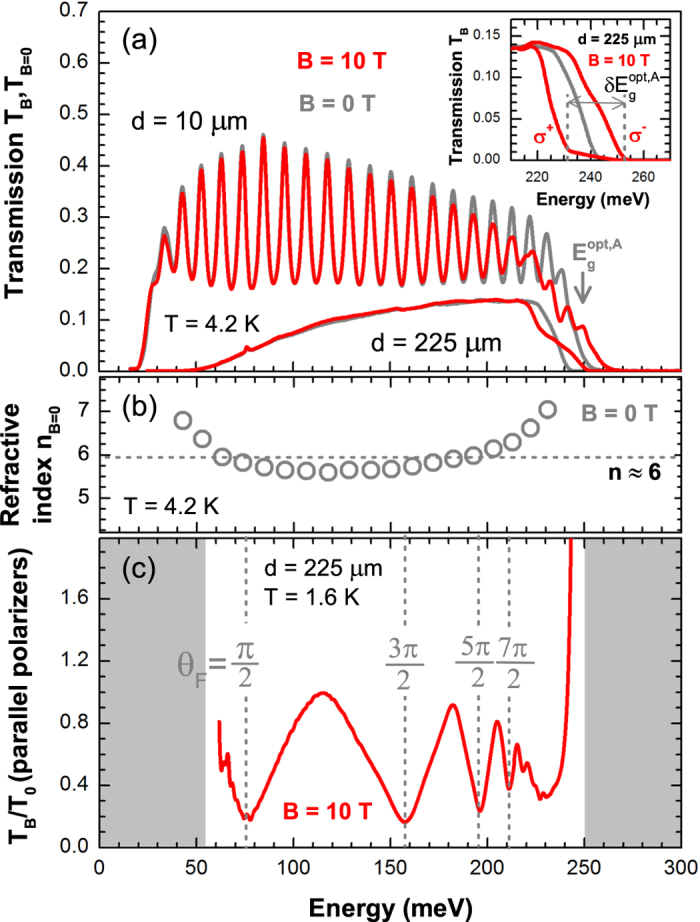
(**a**) Low temperature infrared transmission of the sample A at B = 0 and 10 T measured on free-standing layers with a thickness of 10 and 225 *μ*m. The sample is transparent in the spectral window defined at lower energies by the plasma frequency 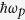 and at higher energies by the interband absorption, implying the optical band gap of 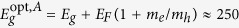 meV due to the Burstein-Moss shift. This transparency window becomes narrower in thicker samples, due to free carrier absorption at low energies and due to the broadening of the interband absorption edge giving rise to non-zero absorption below 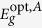. When the magnetic field is applied, the interband absorption edge exhibits strong splitting 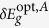, see Eq. 1, when probed by circularly polarized light, see the inset of the part (**a**). The pronounced modulation of the spectrum from the thinner sample are Fabry-Pérot interference fringes, which show high crystalline quality of the Bi_2_Se_3_ bulk specimen and provide us with an estimate of the refractive index plotted in the part (**b**). (**c**) Relative magneto-transmission of the 225-*μ*m-thick free-standing Bi_2_Se_3_ layer prepared from sample A and placed in between two co-linear polarizers. The observed minima correspond to the Faraday rotation angle 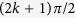 for 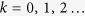

The Faraday rotation measurements have been performed on thin layers of Bi_2_Se_3_ sliced from bulk crystals (A, B and C) with various bulk electron densities 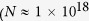, 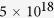 and 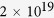 cm^−3^). The prepared specimens with various thicknesses were characterized by infrared transmission at 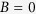. The typical zero-field response observed is illustrated in Fig. 1a, where transmission spectra taken on two free-standing layers prepared from the A crystal (with thicknesses of 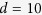 and 225 *μ*m) are plotted. For the 10-*μ*m-thick sample, the transmission window approximatively spans from the plasma frequency 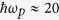 meV up to the interband absorption edge (optical band gap) 
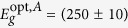
 meV. The difference between 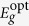 and the energy band gap 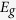, typical of doped degenerate semiconductors, is usually referred to as the Burstein-Moss shift^16^. For sample A, this implies a zero-field Fermi level of 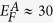 meV, assuming the parameters derived in ref. 17 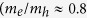 and 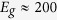 meV).

The pronounced Fabry-Pérot interference pattern observed in the transmission spectrum of the 10-*μ*m-thick specimen, see Fig. 1a, allows us to estimate the refractive index of Bi_2_Se_3_ at photon energies within the window of high transparency, as shown in Fig. 1b, with the averaged value of 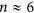. The absolute transmission of the 10-*μ*m-thick layer, 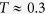, is close to the theoretical value 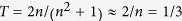 for non-absorbing medium characterized by the refractive index *n*. In the thicker sample, the below-gap absorption is no longer negligible, implying significantly lower absolute transmission 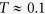 and also noticeably narrower transmission window. The results obtained in transmission measurements on the other two specimens were analogous, providing us with the optical band gaps of 
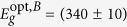
 meV and 
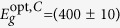
 meV. The declared errors here are mostly due to the variation of the electron density across the bulk crystals.

Interestingly, the application of the magnetic field gives rise to a strong modification of the interband absorption edge of Bi_2_Se_3_. Namely, a splitting with respect to the circular polarization of the probing radiation appears, as shown in the inset of Fig. 1a. This splitting was found to be linear in *B* and the same for all the three investigated crystals: 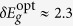 meV/T. In the transmission spectrum taken with non-polarized (or linearly polarized) radiation, this splitting manifests itself as a characteristic step-like profile of the interband absorption edge, see Fig. 1a. This significant difference in interband absorption for circularly polarized light of the opposite helicity is the origin of the strong interband Faraday rotation discussed in this paper.

**Figure 2:**
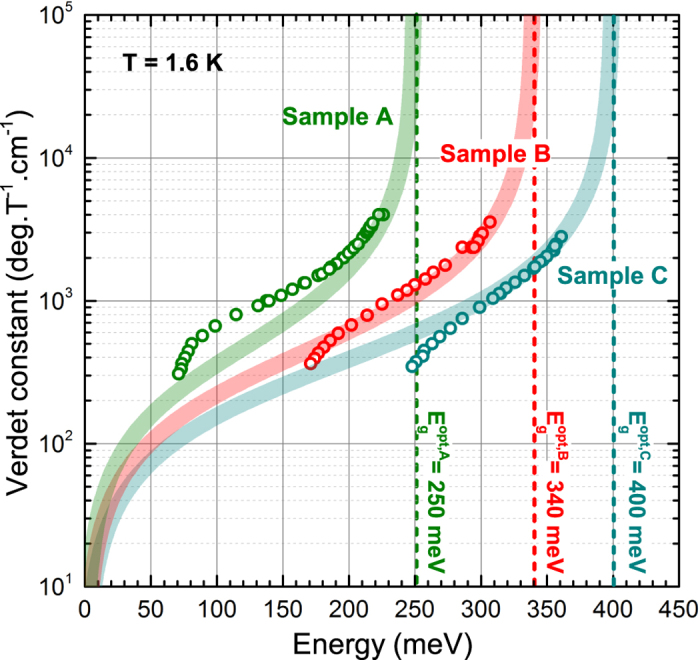
The experimentally determined Verdet constant for samples (A–C) as a function of the photon energy. The theoretical curves have been plotted using Eg. 6, their widths reflect the uncertainty in the determination of the interband absorption edges and sample thicknesses.

The extraordinarily strong Faraday effect can be probed using a simple configuration with the sample placed in between two co-linearly oriented polarizers. The Faraday effect is then manifested by a characteristic modulation of the relative magneto-transmission spectra 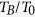, see Fig. 1c. This spectrum has been taken at 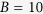 T on the 225-*μ*m-thick layer prepared from the crystal A. The pronounced minima can be easily identified with particular Faraday angles 
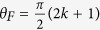
, 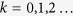, and the corresponding Verdet constants (normalized Faraday angles), 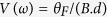, can be calculated from 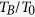 curves measured at various values of *B*. The frequency dependence of the Verdet constant, deduced for samples A, B and C, has been plotted in Fig. 2. Notably, no field-dependence of the Verdet constant has been revealed within the range of the magnetic field applied (up to 13 T).

## Discussion

**Figure 3:**
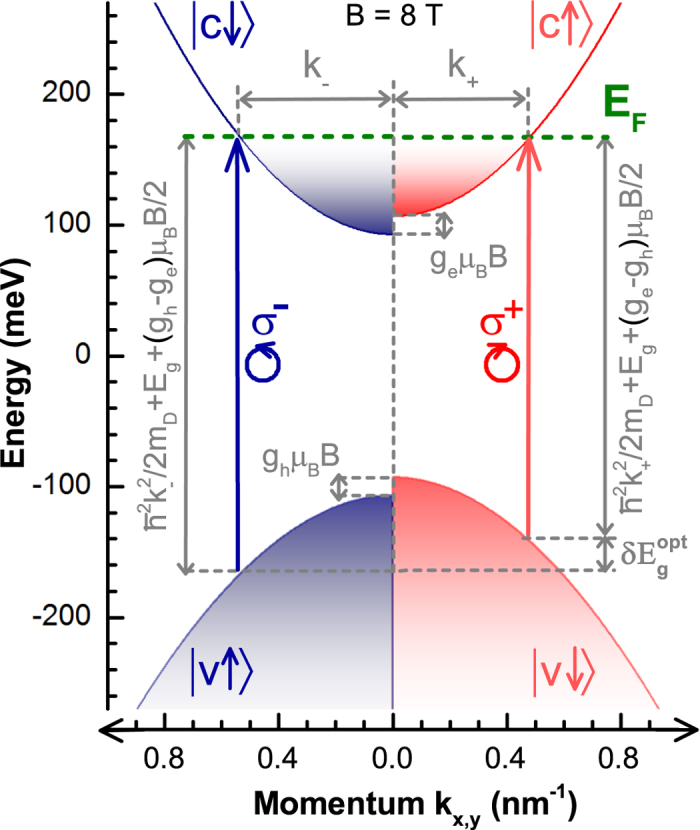
Nearly parabolic conduction and valence bands of Bi_2_Se_3_, around the Γ point of the Brillouin zone, with a significant spin-splitting due to an externally applied magnetic field (*B* = 8 T). The vertical arrows denote the lowest energy interband absorption in right and left polarized radiation, split in energy by 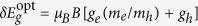. This splitting may be easily derived when we consider that the lowest momenta 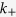 and 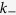, for which interband absorption is allowed in a given circular polarization, satisfy the condition: 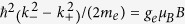. 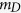 used in formulae in the figure stands for the reduced mass 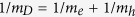, which for Bi_2_Se_3_ equals to the Dirac mass 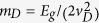.

To account for the observed magneto-optical response let us recall the band structure of Bi_2_Se_3_. Within the past few years, the exact shape of electronic bands in this material has been subject of vast discussions. Nevertheless, Bi_2_Se_3_ is most likely a direct band-gap semiconductor, see, *e.g.*, refs 18,19, which can be well-described by Dirac-type models such as the one proposed in refs 20,21. Our recent magneto-optical study^17^ implies that the conduction and valence bands are nearly parabolic with a high degree of the electron-hole symmetry, see Fig. 3. This electronic band structure can be described using a simplified Dirac-type Hamiltonian for massive particles, which contains only two parameters: the band gap 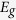 and the velocity parameter 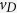. These two parameters provide us with reasonably accurate estimates for the effective masses and *g* factors: 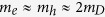 and 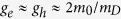, respectively, where 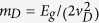 is the Dirac mass.

Large *g* factors in Bi_2_Se_3_ give rise to a specific regime at low magnetic fields, when a pronounced spin-splitting of electronic states appears, but Landau levels are still not well resolved 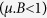, as schematically sketched in Fig. 3. Within this picture of spin-split bands, the interband absorption edge splits with respect to the circular polarization of light:


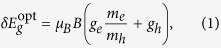


where 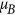 is the Bohr magneton. This formula may be straightforwardly derived, using purely geometrical arguments, see Fig. 3 and its caption.

Notably, due to nearly equal *g* factors of electrons and holes in Bi_2_Se_3_ 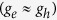, a pronounced splitting of the interband absorption edge (1) only appears in this material when the conduction band is partially filled by electrons (or alternatively the valence band by holes). The observed Faraday rotation, even though primarily related to the circular dichroism of the interband absorption, is thus basically induced by the presence of free conduction-band electrons. However, this effect should be clearly distinguished from the intraband Faraday rotation due to free charge carriers, where the circular dichroism originates in cyclotron motion of particles and related resonant absorption^22^.

Importantly, the splitting (1) does not explicitly depend on the electron density, in agreement with our experiments. The carrier density only determines the saturation field 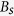, at which a full spin-polarization of electrons is achieved, 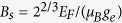, see ref. 23. Above 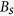, the formula (1) is no longer valid and the splitting saturates at 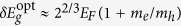 when 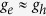. Taking the parameters derived in ref. 17 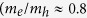, 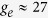 and 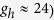 we get 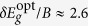 meV/T in very good agreement with the experiment. The saturation field for the lowest doped specimen A should reach 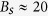 T, well above the magnetic fields applied in the experiments presented here.

The Verdet constant is proportional to the difference between refractive indices for right “+” and left “−” circularly polarized light: 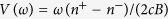, which can be approximated in a weakly absorbing medium by^24^:


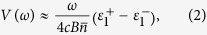


where 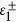 stands for the real part of the dielectric function, 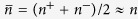 and *c* is the speed of light in vacuum.

When we neglect the contribution from all electronic bands other than the conduction and valence bands and assume the interband matrix elements to be independent of the magnetic field and momentum, 
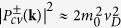
, see ref. 21, the imaginary (dissipative) part of the dielectric function at low magnetic fields and temperatures reads, for a given circular polarization^25^:


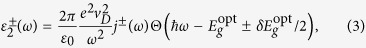


where 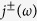 is the joint density of states and 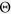 is the Heaviside step function, which describes the low-energy onset of interband absorption at the photon energy of 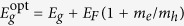.

Assuming strictly equal spin splitting for electrons and holes 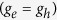 and neglecting the anisotropy of electronic bands, the joint density of states becomes identical for both circular polarizations, 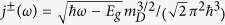, and the imaginary part of the dielectric function (3) takes the form:


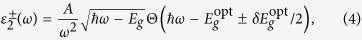


where 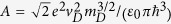.

Using the Kramers-Kronig relations applied to 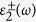 together with Eq. 2, the Verdet constant can be expressed as:


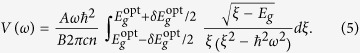


Taking assumption of the full electron-hole symmetry 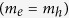, we finally get, in the limit of low magnetic fields 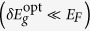, an approximative expression: (*α* is the fine structure constant):


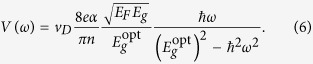


It is worth noting that in the low-field limit, the density of spin-polarized carriers, 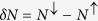, can be approximated as the spin-splitting, 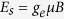, multiplied by the zero-field density of states, 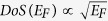. The Faraday angle corresponding to Eq. 6, 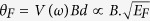, thus becomes directly proportional to 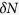. This may resemble the interband Faraday effect due to the Raman spin-flip of electrons bound to In-donors in CdS, reported by Romestain *et al.*^26^, and later on, of free conduction-band electrons in InSb and HgCdTe^27^,^28^. However, the mechanism of the Faraday rotation reported here is different from that considered in refs 26, 27, 28 and is uniquely related to dipole-allowed interband absorption. While in the Raman spin-flip induced Faraday rotation, the angle becomes proportional to the spin splitting of electrons 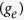, in our case, the resulting rotation is clearly sensitive to the spin splitting of both electrons and holes (both 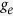 and 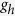 factors), as seen from the initial Eq. 1.

Scaling the photon energy with respect to the optical band gap, 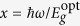, we get the characteristic frequency profile of the interband Faraday rotation: 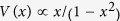, 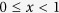. Interestingly, this profile has a considerably simpler form as compared to the expressions derived for and applied to undoped semiconductors^22^,^24^,^29^,^30^. This is due to the match between the electron and hole spin splitting in Bi_2_Se_3_, 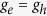, which implies the same joint density of states for both circular polarizations, and therefore, only the finite integration range in the Kramers-Kronig transformation, see Eq. 5. Another simplification, which again comes directly from the Dirac-type Hamiltonian valid for Bi_2_Se_3_, is rather high degree of electron-hole symmetry 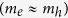. This allows us to express the optical band gap as 
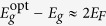
.

We should stress that the formula (6) derived for the Verdet constant in Bi_2_Se_3_ does not contain any tunable parameters. The bulk band gap 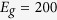 meV and the velocity parameter 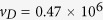 m/s are known from experiments, for instance, from our recent Landau level spectroscopy of thin Bi_2_Se_3_ layers^17^. The refractive index 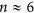 and the optical band gaps 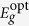 are directly read from transmission spectra of thin specimens at 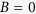, as illustrated for sample A in Fig. 1a,b.

The theoretical curves, calculated using these parameters in Eq. 6, are in good quantitative agreement with the experimentally determined Verdet constant, see Fig. 2. For the crystals B and C, the major deviations appear only at lower energies, where the contribution to the total Faraday rotation arising from the cyclotron resonance absorption^22^ (fully intraband process) is no longer negligible. For the lowest-doped sample A, the spin-splitting becomes, for the studied range of magnetic fields, comparable with the Fermi level, which brings the formula (6) close to the limit of its validity.

Let us now compare our results with the Faraday rotation on other materials, the choice of which remains, in the equivalent spectral range, still limited. Taking the reference at the wavelength 10.6 *μ*m of the CO_2_ laser, the highest specific interband Faraday rotation for non-magnetic materials has been reported for InSb, 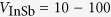 deg/(T.cm), see refs 31 and 32. Interestingly, this semiconductor has a band gap nearly identical to Bi_2_Se_3_. This rotation is at least one order of magnitude lower compared to the value we have found in Bi_2_Se_3_: 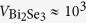 deg/(T.cm), see the Verdet constant at wavelength of 10.6 *μ*m (corresponding to the photon energy of ≈120 meV) for the lowest doped specimen A in Fig. 2. In fact, the observed Verdet constants become comparable to values known for magnetic semiconductors, see, *e.g.*, ref. 33.

The relatively high Verdet constants of Bi_2_Se_3_ invoke the possibility to use it as the active medium in Faraday rotators/insulators although one has to keep in mind the Drude-type absorption on free conduction-band electrons, which lowers the overall transmission. This absorption may be reduced, by decreasing the electron density, nevertheless, this may be a challenging task for the current growth technology of this material. Moreover, this would also lower the saturation field 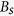.

It is instructive to discuss the implications of our results on other 3D TIs from the same family, such as Bi_2_Te_3_ or Sb_2_Te_3_. Most likely, their bands strongly deviate from the parabolic profiles^34^, which are characteristic of Bi_2_Se_3_, but still they are characterized by similar band gaps, and also, as seen, for instance, from ARPES experiments^35^, similar velocity parameters. Since these materials are described by the same expanded 3D Dirac Hamiltonian^20^,^21^, we may expect the electron and hole *g* factors to roughly follow the simple estimate 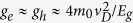 deduced for Bi_2_Se_3_ in ref. 17. This would imply an analogous splitting of the interband absorption edge given by Eq. 1 and equally strong interband Faraday effect in doped TIs.

To conclude, we have probed the Faraday rotation induced by interband excitations in a series of bulk Bi_2_Se_3_ specimens. We show that this effect is at least by an order of magnitude stronger than in other non-magnetic materials. We demonstrate that the particular strength of the effect has its origin in the relativistic-like Hamiltonian applicable to Bi_2_Se_3_ thanks to which electrons and holes behave as massive Dirac particles. A simple formula based on this two-parameter Dirac-type Hamiltonian is derived to describe this phenomenon quantitatively, requiring no tunable parameters. We also predict that similarly strong interband Faraday effect should be present in other 3D topological insulators, in particular in those from the Bi_2_Se_3_ family.

## Methods

The studied Bi_2_Se_3_ crystals have been prepared using the standard Bridgman method. The starting material for growing the single crystals was prepared from the elements Bi and Se of 5 N purity. Polycrystalline material was prepared from a mixture of the elements close to stoichiometry (Bi:Se = 2:3) in silica ampoules evacuated to a pressure of 10^−4^ Pa. The synthesis was carried out at the temperature of 1073 K. A conical quartz ampoule, containing the synthesized polycrystalline material, was then placed in the upper (warmer) part of the Bridgman furnace, where it was remelted. Then it was lowered into a temperature gradient of 80 K/cm (30 K/cm for the sample C) at a rate of 1.3 mm/h. Three bulk crystals, differing in the concentration of conduction-band electrons in the conduction band, have been chosen for this study. They were characterized by approximate electron densities 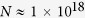, 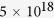 and 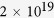 cm^−3^ and denoted as samples A, B and C, respectively.

The prepared single crystals, easily cleavable along the hexagonal planes (0001), were sliced, using a microtome machine to free-standing layers with various thicknesses. All experiments, magneto-transmission and Faraday-angle measurements, were performed in the Faraday configuration with light propagating along the *c* axis of Bi_2_Se_3_. A macroscopic area of the sample (~4 mm^2^) was exposed to the radiation of a globar, which was analysed by a Fourier transform spectrometer and, using light-pipe optics, delivered to the sample placed in a superconducting magnet. The transmitted light was detected by a composite bolometer placed directly below the sample, kept at a temperature of 1.6 or 4.2 K. To measure the Faraday rotation, the specimens were placed in between two co-linearly oriented wire-grid polarizers defined holographically on a KRS-5 substrate. In experiments performed with circularly polarized light, a linear polarizer and a zero-order MgF_2_ quarter wave plate (centered at 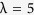 *μ*m) were used.

## Additional Information

**How to cite this article**: Ohnoutek, L. *et al.* Strong interband Faraday rotation in 3D topological insulator Bi_2_Se_3_. *Sci. Rep.* **6**, 19087; doi: 10.1038/srep19087 (2016).
